# NAIL-G: a conceptual best-fit framework for nursing ai literacy and governance anchored in socio-technical safety science

**DOI:** 10.1186/s12912-026-04555-6

**Published:** 2026-04-11

**Authors:** Fatma M. Ibrahim, Ghada Shahrour, Israa A. M. Abuijlan, Amal Mohammed Abdallah, Asmaa Alsuwayed, Abdulkarem Radhi Alanazi

**Affiliations:** 1 Faculty of Nursing, Mansoura University, Mansoura, Egypt; 2 Faculty of Nursing, Jordan University of Science and Technology, Irbid, Jordan; 3 RAK Medical and Health Science University, RAK College of Nursing, RAK, UAE; 4 Department of Management, College of Business, King Saud University, Riyadh, Saudi Arabia; 5 Public Administration Department, College of Business, King Saud University, Riyadh, Saudi Arabia

**Keywords:** Artificial intelligence, Nursing governance, Socio-technical safety, AI literacy, Digital competence, Conceptual framework, Nursing education, Global health policy

## Abstract

**Background:**

Artificial intelligence is reshaping health care delivery and education, but nurses lack discipline-specific guidance that integrates artificial intelligence literacy with socio-technical safety and governance. Existing AI literacy and digital competence models are often generic and do not reflect nursing standards, work systems or responsibilities for patient safety and equity.

**Methods:**

A best-fit framework synthesis and concept development were used to integrate scholarship on artificial intelligence literacy, digital competence, socio-technical safety, nursing competency standards and policy guidance on transparent use of artificial intelligence in scholarly work, with extracted constructs mapped to the AACN Essentials and socio-technical work-system dimensions.

**Results:**

The Nursing Artificial Intelligence Literacy and Governance (NAIL-G) framework comprises five domains covering Foundations & Tools; Quality, Safety & Risk; Professionalism, Ethics & Law; Systems, Equity & Social Impact; and Scholarship, Communication & Governance. It positions artificial intelligence literacy as competency-plus-governance, emphasizing critical judgement, verification, disclosure and equity-aware practice across nursing education, clinical work and scholarly communication.

**Conclusions:**

NAIL-G offers curriculum designers, educators, regulators and clinical leaders a structured way to embed artificial intelligence capabilities, safeguards and governance expectations across nursing practice and education. The framework can guide integration of artificial intelligence-related learning outcomes, assessment tasks and safety checks into curricula, support reflective use of artificial intelligence in clinical reasoning and documentation, and strengthen nurses’ confidence to raise concerns about unsafe or inequitable systems.

## Introduction

Artificial intelligence (AI) tools, including large language models and predictive analytics, are increasingly embedded in clinical workflows, electronic health records, population health management, learning management systems, and scholarly writing platforms. Nurses across the world are already encountering AI when documenting care, triaging patients, planning discharges, monitoring populations, and supporting students. However, the speed of AI diffusion has outpaced discipline-specific literacy, governance, and safety guidance in nursing education and practice.

Terminology: In this paper, we use the term “AI tools” as an umbrella term for software applications that generate, summarize, classify, predict, or recommend content using machine-learning approaches. We distinguish between (i) generative AI (e.g., large language models (LLMs) that generate text, and large multimodal models (LMMs) that generate or interpret combinations of text, images, or other modalities) and (ii) predictive AI/analytics (e.g., machine-learning models that estimate risk or classify patients). Unless otherwise stated, “AI” refers to these clinical, educational, and scholarly applications.

Global organizations, including the World Health Organization (WHO), have called for robust ethical and governance frameworks for AI in health, emphasizing transparency, accountability, equity, and human oversight [1, 2]. Professional bodies similarly stress that AI must augment rather than replace clinical judgment and that human clinicians retain ultimate responsibility for decisions and documentation [3]. Despite these calls, there is limited guidance on how nursing programs, regulators, and institutions should design curricula, policies, and assessment systems that build responsible AI literacy and governance capabilities among nurses.

Existing AI literacy and digital competence frameworks provide valuable starting points [4–7]. Yet most are designed for general citizens or higher education broadly, not for nursing’s distinct socio-technical work systems. Nursing practice involves complex interaction of people, technologies, tasks, physical environments, organizations, and external policy contexts. Socio-technical safety science in health information technology [8, 9] shows that even well-designed tools can create new hazards if they are poorly implemented or used without adequate training, governance, and feedback mechanisms.

In parallel, academic journals, editors, and international councils are developing policies for the ethical and transparent use of AI in scholarly manuscripts. Recent recommendations from the International Committee of Medical Journal Editors (ICMJE), the World Association of Medical Editors (WAME), and the Committee on Publication Ethics (COPE) emphasize that AI tools cannot be listed as authors, that their use must be disclosed, and that human authors remain accountable for accuracy, originality, and integrity [10–12]. Nursing students and faculty must therefore become literate not only in using AI tools, but also in adhering to governance and disclosure expectations across educational and publication contexts.

Taken together, these developments signal the need for a nursing-specific framework that unites AI literacy, socio-technical safety science, professional and ethical standards, and governance requirements. This paper responds to that need by developing NAIL-G, a conceptual best-fit framework for Nursing AI Literacy and Governance.

Novelty claim: NAIL-G extends existing AI literacy and digital competence models by (a) coupling nursing AI literacy with explicit governance expectations (e.g., disclosure, incident learning, curriculum oversight and audit-based indicators), (b) anchoring domains to both nursing competency standards and socio-technical safety science, and (c) providing a structured set of measurable indicators to support curriculum mapping and organizational governance.

## Background and rationale

### AI in nursing and health professions education

AI applications now influence triage systems, risk prediction, staffing optimization, documentation support, simulation-based learning, and formative feedback in health professions education [13]. Generative AI tools can draft care plans, learning objectives, feedback comments, and scholarly text, while predictive models support early warning systems and workload management.

For nursing, these technologies offer opportunities to reduce documentation burden, support decision-making, and personalize learning. However, they also raise concerns about over-reliance, deskilling, data privacy, bias, misinformation, and erosion of trust if AI-generated outputs are inaccurate, opaque, or undisclosed. Nursing, as a profession grounded in ethical practice, human dignity, relational care, and advocacy, requires AI use to be embedded within robust ethical and governance frameworks, not treated as a purely technical skill set [3].

### Limitations of current AI literacy and digital competence frameworks

Recent scholarship has articulated conceptualizations of AI literacy as encompassing knowledge, skills, attitudes, and ethical awareness needed to interact with AI critically and effectively [4, 5]. Digital competence frameworks such as DigComp 2.2 and the Jisc Digital Capabilities Framework extend this perspective by situating AI within broader digital skills for citizens and higher-education staff [6, 7].

While these frameworks are valuable, they have limitations for nursing. First, they are not explicitly anchored in nursing standards such as the American Association of Colleges of Nursing (AACN) Essentials or other national competency frameworks [14]. Second, they do not systematically integrate socio-technical safety considerations, including how AI interacts with workflows, organizational structures, and regulatory environments. Third, governance mechanisms such as disclosure policies, incident reporting, risk controls, and curriculum oversight, are often peripheral or absent.

As a result, educators seeking to design AI-related learning outcomes and assessments for nursing programmes may struggle to connect general AI literacy models to profession-specific expectations, patient safety obligations, and policy requirements. There is a clear need for a framework that is discipline-specific, safety-oriented, and governance-aware.

### Socio-technical safety and governance gaps

Health information technology safety research demonstrates that patient harm can arise from the interaction of technology with people, tasks, teams, organizations, and environments, rather than from software alone [8, 9]. Socio-technical models such as SEIPS and the eight-dimension framework of Sittig and Singh emphasize the importance of designing, monitoring, and governing technology within an integrated system.

Generative and predictive AI share many of the same socio-technical risks as electronic health records and decision-support systems, including alert fatigue, workarounds, documentation errors, inequities, and new forms of cognitive burden. They add further challenges related to hallucinations, non-deterministic outputs, data provenance, privacy, and the potential amplification of historical biases [1, 2]. Without governance structures that address these system-level risks, AI literacy focused only on individual skills is insufficient.

Journal and editorial policies on AI use also function as governance mechanisms. Guidance from ICMJE, WAME, and COPE calls for transparency, attribution, and human accountability, creating expectations for how students, educators, and researchers use AI in scholarly work [10–12]. Nursing programs therefore need frameworks that link classroom teaching, clinical decision-making, and scholarly communication to consistent governance principles.

### Aim

The aim of this paper is to develop a conceptual framework for Nursing AI Literacy and Governance (NAIL-G) that:

- Integrates existing AI literacy, digital competence, nursing standards, and socio-technical safety frameworks.
- Positions AI literacy as competency-plus-governance, rather than as technical skills alone.
- Provides actionable domains and indicators that can guide curriculum design, assessment, and governance in nursing programmes.
- Offers a basis for future empirical evaluation of AI literacy and governance interventions in nursing education and practice.

## Methods: best-fit framework synthesis with concept development

### Design

This paper reports on a conceptual study using a best-fit framework synthesis combined with concept-development strategies drawn from nursing scholarship. Best-fit framework synthesis is useful when multiple candidate models exist, and the goal is to integrate them into a context-specific structure that offers high explanatory and practical fit. Concept development methods, such as those described by Walker and Avant and by Rodgers, support clarification of attributes, antecedents, and consequences of key constructs, and refinement of domain boundaries.

### Data sources and framework selection

Search approach: We used a purposive, iterative searching strategy to identify candidate frameworks and guidance relevant to AI literacy, advanced digital competence, socio-technical safety, nursing competency standards, and AI governance in scholarly work. Bibliographic searches were conducted in PubMed/MEDLINE, CINAHL, Scopus, Web of Science, and ERIC, supplemented by targeted searching for socio-technical and health IT safety concepts. Grey literature and policy sources were identified through targeted website searches of major organisations (e.g., WHO, ANA, AACN, ICMJE, WAME, COPE) and citation-chaining from key papers.

Time window and key terms: Searches were limited to sources published between January 2010 and December 2025. Search strings combined terms for AI and literacy/competence (e.g., “artificial intelligence” OR “generative AI” OR “machine learning” AND “literacy” OR “competence” OR “capability”) with nursing/education and safety/governance concepts (e.g., nursing, health professions education, socio-technical, safety, risk, ethics, equity, governance, disclosure, publication policy). Searches were conducted iteratively and updated through December 2025.

We drew on five main groups of data sources:

1. AI literacy and AI-in-education frameworks and reviews (e.g. [4, 5, 13]).
2. Digital competence models, including DigComp 2.2 and Jisc Digital Capabilities [6, 7].
3. Nursing competency standards, with particular emphasis on the AACN Essentials for professional nursing education [14].
4. Socio-technical and health IT safety frameworks, especially SEIPS and the socio-technical model of Sittig and Singh [8, 9].
5. Editorial and governance policies on AI use in scholarly manuscripts from ICMJE, WAME, COPE, and WHO guidance on AI ethics and governance [1, 2, 10–12].

Frameworks were included if they explicitly addressed AI or advanced digital competence, were applicable to higher education or health professions, or provided governance and safety guidance relevant to AI-supported work.

Because the searching strategy was purposive and iterative rather than a systematic review, we did not maintain PRISMA-style record counts. To support transparency and reproducibility, we provide an inventory of the final included set of core frameworks and guidance documents used for coding and mapping (*n* = 14) in Table 1.

**Table 1:** Included frameworks and guidance documents used for construct extraction and what each contributed to NAIL-G

Source group	Framework / guidance document	Key constructs extracted	Primary contribution to NAIL-G
AI ethics/governance (health)	WHO Ethics and governance of AI for health (2021) [1]	Ethical principles; governance actions; accountability; transparency; human oversight; equity	Domains 2–4; governance framing and equity indicators
AI ethics/governance (health)	WHO Ethics and governance of AI for health: guidance on large multimodal models (2025) [2]	Model capability/limit considerations for LMMs; risk controls; transparency; safety monitoring	Domains 1–2 and 4; terminology and risk controls for LLM/LMM use
Professional nursing guidance	American Nurses Association position statement on ethical use of AI (2022) [3]	Professional responsibilities; accountability; advocacy; ethical practice; augmentation vs. replacement	Domains 2–4; nursing-specific ethical framing and accountability
AI literacy scholarship	Ng et al. AI literacy review (2021) [4]	AI literacy components (knowledge, skills, attitudes); critical evaluation; ethical awareness	Domain 1 and 3; foundational literacy competencies
AI literacy scholarship	Long and Magerko AI literacy competencies (2020) [5]	Competency set for AI literacy; user interaction; evaluation of outputs	Domain 1; competency language for tool use and verification
Digital competence	DigComp 2.2 (2022) [6]	Digital information skills; content creation; safety; problem solving; AI examples	Domains 1–2 and 5; digital-capability scaffolding and assessment ideas
Digital capability (HE)	Jisc Digital Capabilities (n.d.) [7]	Digital identity; information literacy; communication; wellbeing and safety	Domains 1 and 5; communication/provenance and capability framing
Socio-technical safety	SEIPS 2.0 (2013) [8]	Work system components; processes; outcomes; interactions across people/tasks/tools/organization/environment	Domain 2 and 4; system-based safety lens and work-system dimensions
Socio-technical safety	Sittig and Singh socio-technical model (2010) [9]	Health IT safety dimensions; implementation/monitoring; sociotechnical interactions	Domain 2; safety governance and monitoring practices
Scholarly governance	ICMJE recommendations on authorship and AI acknowledgement (2024) [10]	AI tools not authors; disclosure expectations; human accountability	Domain 5; disclosure, accountability and manuscript governance
Scholarly governance	WAME recommendations on chatbots/genAI and manuscripts (2023) [11]	Transparency; accountability; responsible use and reporting	Domain 5; disclosure and integrity practices
Scholarly governance	COPE position statement on authorship and AI tools (2023) [12]	Authorship boundaries; disclosure; plagiarism/integrity risks	Domain 5; integrity and governance safeguards
Health professions education	National Academies workshop on AI in HPE (2023) [13]	Educational opportunities/risks; curricular considerations; assessment approaches	Domains 1–2 and 5; implementation/assessment considerations
Nursing competency standards	AACN Essentials (2021) [14]	Nursing competency domains; professional formation; informatics/technology expectations	Cross-cutting anchor; mapping of NAIL-G to nursing standards

Inclusion and exclusion criteria: Sources were included if they (i) proposed AI literacy or advanced digital competence domains/competencies, (ii) provided nursing competency standards relevant to technology-enabled practice (e.g., AACN Essentials), (iii) articulated socio-technical or health IT safety dimensions applicable to AI-enabled workflows (e.g., SEIPS; Sittig & Singh), or (iv) provided governance/ethical guidance on AI use in healthcare or scholarly publishing (e.g., WHO; ICMJE/WAME/COPE). We excluded primary algorithm-development papers without a literacy, competency, safety, equity, or governance component; sources that were purely technical with no educational or practice implications; and frameworks with minimal transferability to healthcare. Non-English sources were not included due to feasibility constraints.

Selection process: Screening and selection were led by FI. Formal disagreement procedures were not applicable because screening was conducted by a single reviewer; however, the included-source list and resulting domain structure were reviewed by the co-authors for completeness and face validity. To enhance coverage and reduce the risk of omission, we used backward and forward citation-chaining from key papers, purposive inclusion of authoritative policy sources, and cross-checking against the five pre-specified source groups (AI literacy, digital competence, nursing standards, socio-technical safety, and publication/governance policies).

### Mapping and synthesis procedures

Synthesis steps (extraction and coding): Constructs (e.g., domains, competencies, safeguards, and governance recommendations) were extracted into a structured matrix capturing the construct label, definition, source, and context (education, practice, or governance). We then mapped constructs deductively to two a priori anchors: (i) the AACN Essentials domains and (ii) socio-technical work-system dimensions (people, tasks, tools and technologies, physical environment, organization, and external environment). Constructs that did not fit these anchors were coded inductively as additional subcodes. Extraction and initial mapping were performed by FI; the extraction matrix and domain assignments were reviewed by the co-authors, and suggested revisions were discussed until consensus was reached.

**Fig. 1:**
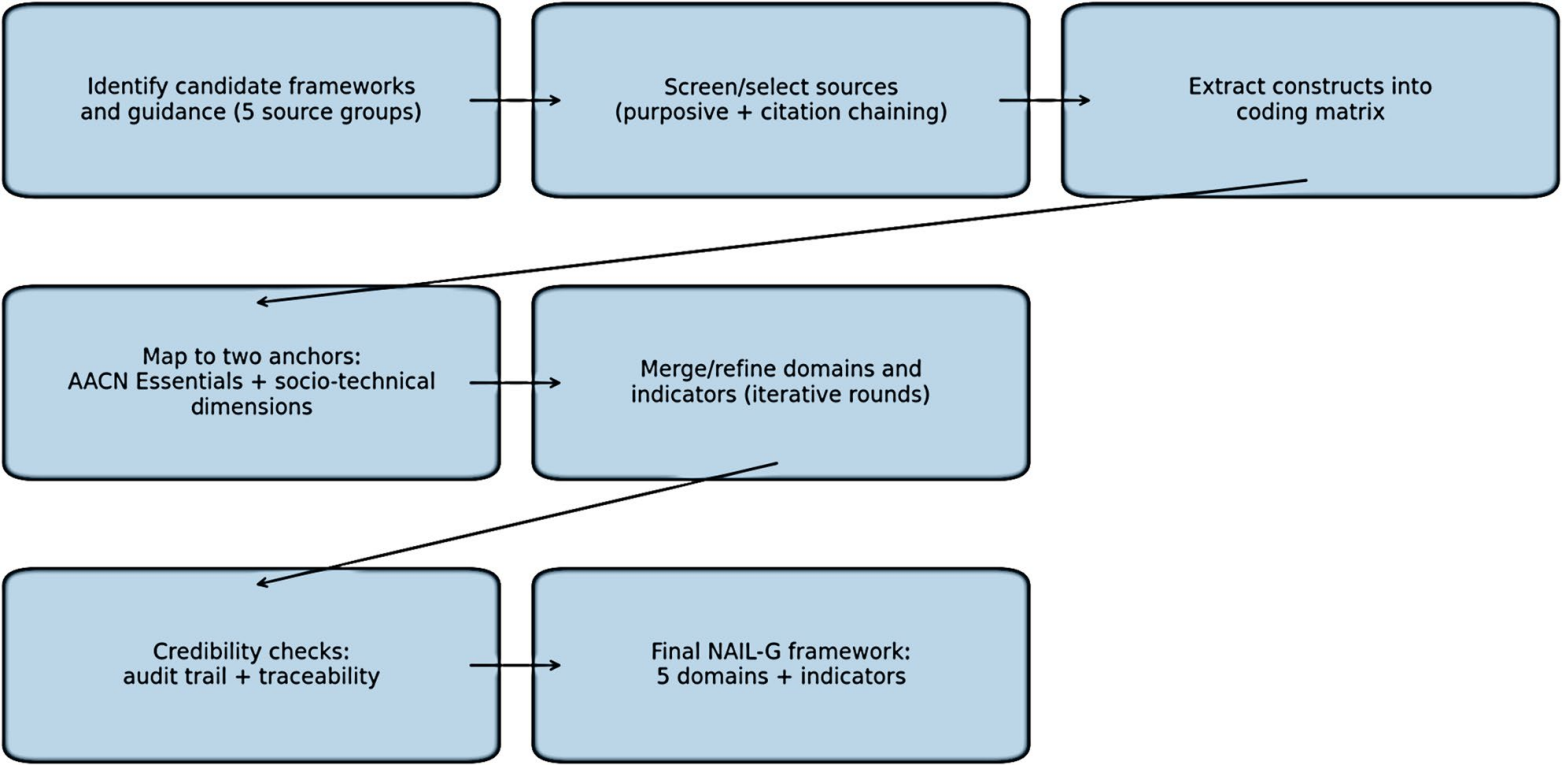
Workflow for best-fit framework synthesis and concept development used to develop NAIL-G

Synthesis steps (merging and finalising domains): Codes were compared for conceptual overlap and merged into candidate NAIL-G domains. Domain boundaries were refined through iterative rounds of review (revisiting the coding matrix, checking coherence of domain labels, and ensuring that each competency statement and indicator aligned with at least one source). Iteration stopped when additional sources did not yield substantively new constructs and when all constructs could be accommodated without creating additional domains. The overall workflow for the best-fit framework synthesis and concept development is shown in Fig. 1.

### Concept development

Using concept-development strategies, we clarified the defining attributes, antecedents, and consequences of each emerging domain. Attributes captured what it means to be literate and governed in each area (e.g., understanding model limitations, documenting AI use, applying safety checks, recognizing bias). Antecedents included organizational policies, curriculum structures, technology availability, and faculty readiness. Consequences included safer AI-supported practice, improved quality of documentation and scholarship, enhanced equity, and strengthened public trust.

We also developed illustrative teaching and governance cases that link domains to observable behaviors and assessment indicators, such as artifact audits, disclosure statements, safety checklist completion, and equity impact analyses.

### Quality and credibility checks

To enhance trustworthiness, we maintained an audit trail documenting search decision, inclusion/exclusion judgements, and the rationale for coding and domain boundary decisions. Reflexive memoing was used to record assumptions and to justify revisions made during iterative synthesis and concept development.

As an internal credibility check, we conducted traceability checks by re-examining the extraction matrix to ensure that each final domain, competency statement, and indicator could be linked to at least one included source and that the full set of domains collectively covered both the AACN Essentials and core socio-technical dimensions.

### Rationale for chosen methods

The combination of best-fit framework synthesis and concept development was selected for three reasons. First, it accommodates the multiplicity of existing AI, digital competence, and safety frameworks rather than privileging a single model. Second, it is well suited to practice-oriented disciplines such as nursing where theory must be grounded in real-world systems. Third, it enables explicit attention to governance and safety, avoiding narrow or technicist views of AI literacy.

## Results: the NAIL-G framework

### Overview

The synthesis resulted in the Nursing AI Literacy and Governance (NAIL-G) framework, comprising five interrelated domains:

1. Foundations & Tools.
2. Quality, Safety & Risk.
3. Professionalism, Ethics & Law.
4. Systems, Equity & Social Impact.
5. Scholarship, Communication & Governance.

Each domain includes suggested competencies and potential indicators that can guide curriculum development, assessment, and governance. Though distinct for clarity, the domains are synergistic and should be implemented as an integrated system of capabilities and controls. An illustrative mapping of the NAIL-G domains to socio-technical work-system dimensions is provided in Table 2.

**Table 2:** NAIL-G domains mapped to socio-technical work-system dimensions (illustrative alignment)

NAIL-G domain	People	Tasks	Tools / technology	Physical environment	Organization	External environment
1. Foundations & Tools	AI concepts; critical appraisal; prompt literacy; confidence to question outputs	Select appropriate use cases; verify outputs; document inputs and checks	LLMs/LMMs and predictive tools; tool selection; privacy-safe use	Point-of-care constraints; device access; interruption-prone settings	Approved tool lists; training; access controls; acceptable-use rules	Vendor terms; regulatory constraints; evolving standards
2. Quality, Safety & Risk	Situational awareness; escalation behaviors; interprofessional collaboration	Pre-use/post-use safety checks; verification against guidelines; incident reporting	Decision support, documentation aids; monitoring dashboards; safety checklists	Workflow realities (alarms, handoffs); bedside documentation constraints	Implementation governance; risk management; learning systems; feedback loops	Accreditation/regulatory safety expectations; reporting requirements
3. Professionalism, Ethics & Law	Ethical reasoning; accountability; integrity; patient advocacy	Disclosure; consent/confidentiality decisions; manage conflicts of interest	Privacy settings; data minimization; IP-aware use of tools	Confidential spaces for documentation; secure device use	Codes of conduct; academic integrity policies; legal/compliance support	Privacy law; professional regulation; IP/copyright; national guidance
4. Systems, Equity & Social Impact	Equity awareness; cultural humility; advocacy for underserved groups	Equity impact assessments; subgroup performance review; mitigation planning	Bias auditing; calibration checks; multilingual patient education safeguards	Digital divide; language access; accessibility supports in care settings	Procurement criteria; monitoring for disparities; community feedback mechanisms	Population context; policy priorities; social determinants; public trust
5. Scholarship, Communication & Governance	Scholarly integrity; transparency norms; peer appraisal skills	Provenance and reproducibility notes; AI-use statements; peer review governance	Reference managers; plagiarism checks; AI-assisted writing tools with logging	Secure research environments; access to approved platforms	Research integrity offices; curriculum committees; governance dashboards and audits	Journal/editorial policies; funder requirements; publication ethics guidance

### Domain 1: foundations & tools

Domain 1 focuses on foundational AI literacy and tool use. It encompasses the ability to:

- Explain core AI concepts, such as data, training, inference, model architecture, uncertainty, and hallucination.
- Describe how generative and predictive AI tools work at a practical level, including their strengths and limitations.
- Select appropriate tools for tasks such as drafting notes, summarizing evidence, or supporting communication, while recognizing when AI is inappropriate or unsafe.
- Craft and iteratively refine prompts; interpret outputs critically; and verify information against trusted sources.
- Apply basic data protection and privacy principles, such as avoiding the entry of identifiable patient data into non-approved tools.

Potential measurable indicators (examples) include: (i) mean score on a short assessment of core AI concepts and limitations (e.g., training data, uncertainty, hallucination); (ii) percentage of learning artefacts that include documented prompts/inputs and a verification note; and (iii) percentage of students correctly applying institutional data-protection rules in scenario-based assessments.

### Domain 2: quality, safety & risk

Domain 2 emphasizes safe adoption and use of AI within nursing’s socio-technical work systems. Key competencies include:

- Conducting pre-use checks (e.g., confirming the purpose of the tool, appropriate use cases, and organizational approval status).
- Applying post-use verification, such as checking AI-generated recommendations against clinical guidelines, policies, and patient data.
- Documenting AI involvement in clinical and educational artefacts, where appropriate.
- Escalating concerns and participating in incident learning when AI-related hazards or near misses are identified.
- Collaborating with interprofessional teams to design, monitor, and improve AI-enabled workflows.

Anchored in SEIPS and the socio-technical model of Sittig and Singh [8, 9], potential measurable indicators (examples) include: (i) percentage of AI-assisted clinical or educational artefacts with a completed pre-use and post-use safety checklist; (ii) percentage of AI-generated recommendations that are independently verified against clinical guidelines and patient data (with verification documented); (iii) number of AI-related incident or near-miss reports per quarter and time-to-mitigation; and (iv) audit rate of AI-related documentation errors detected in simulated or real notes.

### Domain 3: professionalism, ethics & law

Domain 3 addresses ethical, legal, and professional responsibilities in AI use. Competencies include:

- Applying nursing codes of ethics and professional standards to AI-supported practice and scholarship.
- Ensuring transparency and honesty in the use of AI, particularly in assignments, clinical documentation, and manuscripts.
- Protecting confidentiality, intellectual property, and sensitive data when using AI tools.
- Avoiding over-reliance on AI outputs and maintaining independent clinical reasoning and scholarly judgment.
- Recognizing and responding to bias, misinformation, and conflicts of interest in AI systems and outputs.

Potential measurable indicators (examples) include: (i) percentage of assignments or manuscripts containing a complete AI-use disclosure statement (tool, purpose, verification steps, and human accountability); (ii) number of integrity or policy breaches related to undisclosed AI use, plagiarism, or privacy violations per term; (iii) pass rate on scenario-based assessments of confidentiality, consent, and intellectual property when using AI; and (iv) proportion of artefacts free of fabricated citations or unverifiable sources on audit.

### Domain 4: systems, equity & social impact

Domain 4 expands the focus beyond individual users to consider system-level, equity, and societal impacts of AI. Competencies include:

- Identifying potential algorithmic biases and distributional harms, particularly for marginalized or under-served populations.
- Interpreting performance metrics (e.g., sensitivity, specificity, calibration) and understanding their implications for subgroups.
- Critically appraising the data sources, design, and deployment contexts of AI tools, including whose data are represented and whose may be missing.
- Advocating for equitable, culturally sensitive deployment and ongoing monitoring of AI systems within organizations and health systems.
- Reflecting on the broader social, economic, and environmental implications of AI adoption in healthcare and education.

Concrete examples include: (a) triage or deterioration-risk prediction models that under-predict risk for some under-served groups when training data encode historic access gaps, potentially delaying escalation of care; and (b) generative AI producing patient-education materials that are inaccurate, culturally insensitive, or inappropriate for a patient’s language and health literacy, which may undermine informed decision-making and trust.

Potential measurable indicators (examples) include: (i) number of AI tools or projects with a documented equity impact assessment; (ii) proportion of evaluations that report subgroup performance (e.g., sensitivity/specificity or calibration by age, sex, language, disability status, or other locally relevant characteristics); (iii) percentage of AI-generated patient-education materials meeting readability and cultural-safety checks; and (iv) number of bias or equity concerns raised and addressed through governance pathways. This domain aligns with WHO guidance on ethics and governance of AI for health [1, 2].

### Domain 5: scholarship, communication & governance

Domain 5 focuses on AI use in scholarly communication and the governance structures that support responsible practice. Competencies include:

- Communicating AI-assisted work with clear provenance, versioning, and reproducibility notes.
- Aligning with journal, institutional, and regulatory policies on AI use, authorship, plagiarism, and research integrity [10, 11].
- Preparing AI-use statements that specify the tools used, purposes, verification steps, and human accountability.
- Participating in curriculum and policy governance processes, such as committees that define acceptable AI use in programmes and monitor quality.
- Engaging in peer appraisal of AI-assisted work, using rubrics and audit tools to support consistent standards.

Potential measurable indicators (examples) include: (i) percentage of scholarly artefacts that include an AI-use statement with provenance (tool name/version, purpose, and verification steps); (ii) percentage of manuscripts compliant with journal and institutional policies (e.g., no AI authorship, transparent disclosure); (iii) proportion of assessed artefacts with reproducibility notes sufficient for another reader to repeat the AI-assisted step; and (iv) number of curriculum or policy governance actions related to AI per year (e.g., policy updates, rubric adoption, training completion).

### How NAIL-G extends existing frameworks

NAIL-G builds upon and extends existing AI literacy and digital competence frameworks by:

- Explicitly integrating socio-technical safety science (SEIPS; Sittig & Singh) into AI literacy for nursing.
- Anchoring competencies in nursing standards such as the AACN Essentials and ethical position statements.
- Adding governance structures, indicators, and journal-policy literacy to traditional literacy domains.
- Highlighting equity, system-level impact, and advocacy as core components of AI literacy, rather than optional add-ons.
- Connecting classroom learning, clinical practice, and scholarly communication under a common set of competencies and governance expectations.

## Implementation and evaluation

### Curriculum mapping

NAIL-G can be used as a blueprint for curriculum mapping in undergraduate and postgraduate nursing programs. Educators can map each domain and competent to existing courses, clinical placements, and assessment activities, identifying gaps and redundancies. For example, introductory courses may focus on foundational concepts (Domain 1), while advanced courses address safety, equity, and governance (Domains 2–5).

Programs can define level-appropriate milestones, such as basic awareness at early stages, applied practice during clinical rotations, and leadership and governance capabilities at advanced levels. Alignment with national or regional competency frameworks ensures that AI literacy and governance are not detached from broader professional formation.

### Assessment indicators and tools

Assessment of NAIL-G can combine multiple components:

- AI literacy scales and attitude measures aligned with Domains 1 and 3.
- Artifact audits of assignments, clinical documentation exercises, and scholarly outputs, focused on reference fidelity, provenance notes, safety reflections, and disclosure quality.
- Safety and equity checklists completed alongside AI-assisted tasks.
- Rubrics that explicitly evaluate appropriate scope and limits of AI use, ethical reasoning, and governance compliance.

Such a multi-component assessment strategy allows programmes to track not only knowledge acquisition, but also behaviours and practices that reflect safe, ethical, and equitable AI use.

### Pilot evaluation design

A practical evaluation approach might involve embedding NAIL-G-informed learning activities into a course or module over six to eight weeks. Students could complete a pre- and post-intervention AI literacy scale; submit AI-assisted artefacts accompanied by disclosure statements and safety/equity checklists; and provide feedback on feasibility and perceived learning.

Primary outcomes could include changes in literacy scores, improvements in artifact quality (e.g., citation accuracy, clarity of disclosures), and completion rates for safety and equity checklists. Secondary outcomes might involve faculty workload, acceptability, and perceived preparedness of students to engage with AI in practice. Over time, programmes could track longitudinal changes across cohorts to inform continuous improvement.

## Illustrative teaching and governance cases

To support practical implementation, three illustrative cases demonstrate how NAIL-G domains can be translated into teaching and governance activities.

### Case 1

**Clinical reasoning with AI-supported virtual patients**.

Students interact with an AI-driven virtual patient to gather history, interpret findings, and formulate nursing diagnoses and care plans. They must verify clinical information against guidelines, document AI involvement, complete a safety checklist, and reflect on any potential biases or limitations in the AI’s responses. Assessment includes communication and reasoning rubrics, as well as artifact audits of documentation and reflection.

### Case 2

**Evidence brief generation with provenance**.

Students use an AI assistant to draft initial evidence brief on a clinical question, then hand-verify all references, add provenance and reproducibility notes, and write a clear AI-use statement. Assessment focuses on accuracy of references, adequacy of verification steps, and the quality of disclosure and reflection on AI’s role.

### Case 3

**Equity and system impact analysis**.

Students analyze a documented case where an algorithm or AI system produced inequitable outcomes. They identify mechanisms of bias, stakeholders affected, and potential mitigations at the levels of data, model, workflow, and governance. Assessment criteria emphasize depth and accuracy of analysis and practicality of proposed interventions, linked explicitly to NAIL-G domains.

## Discussion

### Conceptual contribution

NAIL-G contributes to the growing discourse on AI in health professions education by explicitly reframing AI literacy for nursing as competency-plus-governance. This perspective moves beyond individual user skills to encompass socio-technical safety, equity, and institutional responsibility. By integrating multiple frameworks from AI literacy, digital competence, nursing standards, and safety science, NAIL-G offers a coherent structure tailored to nursing’s realities.

The framework also addresses a current gap in nursing scholarship by connecting AI use in clinical practice, education, and scholarship under shared ethical and governance principles. As journal and institutional policies on AI continue to evolve, such an integrated approach can support coherent teaching, policy making, and quality improvement.

### Implications for global nursing

For global nursing, NAIL-G offers a flexible yet principled framework that can be adapted across diverse educational systems, regulatory environments, and resource settings. Low- and middle-income countries may focus initially on foundational literacy, safety, and governance of a small number of tools, while high-income contexts may engage more deeply with advanced analytics and organizational governance structures. In all settings, the emphasis on equity and advocacy supports nursing’s longstanding commitments to social justice and human rights.

NAIL-G can also inform continuing professional development and lifelong learning, helping nurses, nurse educators, and nurse leaders update their competencies as AI technologies and policies evolve. Professional associations and regulatory bodies may use the framework to guide position statements, competency standards, and accreditation requirements.

### Policy and governance implications

At the policy level, NAIL-G highlights the importance of aligning AI adoption strategies with nursing workforce development, patient safety goals, and ethical guidelines. Ministries of health, health systems, and educational institutions can use the framework to ensure that AI implementation plans include clear roles for nursing, adequate training, and mechanisms for monitoring impact and unintended consequences.

The governance aspects of NAIL-G encourage the establishment of interdisciplinary committees that include nursing voices in decisions about AI selection, deployment, oversight, and decommissioning. The framework also supports alignment with international guidance from WHO and global editorial bodies on ethics, governance, and transparency.

### Equity and safety considerations

By explicitly incorporating systems, equity, and social impact as a domain, NAIL-G foregrounds concern that are sometimes marginal in AI literacy discussions. It encourages nurses to question whose needs are prioritized in AI design and deployment, whose data are represented, and how harms and benefits are distributed. This orientation complements existing global initiatives to address inequities in digital health and AI.

Safety considerations are similarly central. NAIL-G embeds safety checks, verification practices, incident learning, and governance mechanisms within AI literacy, reinforcing that safe practice is not optional or separate from competence. This integrated view supports nursing’s responsibility to protect patients, families, and communities in the face of rapidly evolving technologies.

## Implications for nursing & health policy

Nursing organizations and regulators can use the Nursing Artificial Intelligence Literacy and Governance framework to clarify expectations for competence, accountability and oversight when artificial intelligence is introduced into education, practice and scholarly work. Mapping the framework to existing standards, scopes of practice and accreditation requirements would support coherent guidance for curricula, continuing professional development and role descriptions for nurses and nurse educators.

At wider health system and policy levels, the framework can inform strategies for responsible artificial intelligence adoption, including investment in workforce development, governance committees with nursing representation and mechanisms for monitoring safety, equity and quality. Explicit attention to socio-technical safety, transparency and equity in policy documents can help ensure that artificial intelligence tools strengthen, rather than undermine, person-centered care, professional judgement and public trust in nursing and health services.

## Limitations

This paper reports a conceptual framework rather than empirical evaluation. The development of NAIL-G relied on purposive selection and synthesis of existing frameworks and guidance; although efforts were made to include diverse and influential sources, some relevant models may not have been included. In addition, the framework has not yet been tested in specific educational programs or practice settings, and its feasibility, reliability, and impact remain to be determined.

Most source frameworks originated from high-income countries, which may limit transferability to some contexts. Future work should involve co-design with stakeholders from diverse regions, including students, practicing nurses, educators, and policymakers, to refine and contextualize NAIL-G for local needs.

## Future directions

Future research should empirically evaluate NAIL-G and its indicators in different nursing education and practice settings. Priorities include:

- Adapting and validating AI literacy and attitude scales mapped to NAIL-G domains.
- Developing and testing artifact audit tools, safety and equity checklists, and governance indicators for reliability and usability.
- Examining the impact of NAIL-G-informed curricula on students’ knowledge, skills, behaviors, and confidence in using AI safely and ethically.
- Exploring how NAIL-G can inform organizational policies, accreditation standards, and regulatory guidance for nursing and allied professions.

Cross-disciplinary collaborations with computer science, informatics, ethics, and health policy will be essential to ensure that NAIL-G remains responsive to technological and regulatory developments.

## Conclusion

NAIL-G offers a nursing-specific, socio-technical, and governance-oriented conceptual framework for AI literacy. By articulating five interlocking domains, Foundations & Tools; Quality, Safety & Risk; Professionalism, Ethics & Law; Systems, Equity & Social Impact; and Scholarship, Communication & Governance the framework provides educators, leaders, and policymakers with a structured basis for curriculum design, governance, and evaluation. NAIL-G’s distinctive contribution lies not only in specifying what nurses should know and do with AI, but also in clarifying how programs and organizations can govern AI use to protect safety, equity, and trust. Empirical evaluation of NAIL-G is now required to establish its feasibility, reliability, and impact on learner and system outcomes.

## Data Availability

Not applicable. No datasets were generated or analysed for this conceptual study.
